# Identification of novel neuroendocrine-specific tumour genes

**DOI:** 10.1038/sj.bjc.6604565

**Published:** 2008-09-30

**Authors:** E Hofsli, T E Wheeler, M Langaas, A Lægreid, L Thommesen

**Affiliations:** 1 Department of Cancer Research and Molecular Medicine, Faculty of Medicine, Norwegian University of Science and Technology, Trondheim, Norway; 2 Oncology Unit, St Olavs Hospital HF, Trondheim, Norway; 3 Department of Mathematical Sciences, Norwegian University of Science and Technology, Trondheim, Norway; 4 Department of Food and Medical technology, Sør-Trøndelag University College, Trondheim, Norway

**Keywords:** neuroendocrine tumours, gene expression, microarray, neuroendocrine markers, cell lines

## Abstract

Neuroendocrine tumours (NETs) comprise a heterogenous group of malignancies with an often unpredictable course, and with limited treatment options. Thus, new diagnostic, prognostic, and therapeutic markers are needed. To shed new lights into the biology of NETs, we have by cDNA transcript profiling, sought to identify genes that are either up- or downregulated in NE as compared with non-NE tumour cells. A panel of six NET and four non-NET cell lines were examined, and out of 12 743 genes examined, we studied in detail the 200 most significantly differentially expressed genes in the comparison. In addition to potential new diagnostic markers (*NEFM*, *CLDN4*, *PEROX2*), the results point to genes that may be involved in the tumorigenesis (*BEX1*, *TMEPAI*, *FOSL1*, *RAB32*), and in the processes of invasion, progression and metastasis (*MME*, *STAT3*, *DCBLD2*) of NETs. Verification by real time qRT–PCR showed a high degree of consistency to the microarray results. Furthermore, the protein expression of some of the genes were examined. The results of our study has opened a window to new areas of research, by uncovering new candidate genes and proteins to be further investigated in the search for new prognostic, predictive, and therapeutic markers in NETs.

Neuroendocrine (NE) tumours (NETs) belong to a heterogenous group of neoplasms arising from malignant transformation of various types of NE cells (Falkmer, 1993; Wick, 2000; DeLellis, 2001; Hofsli, 2006). Although the majority of NETs are rather slow growing, their biology is often unpredictable, making their management a great challenge (Stephenson, 2006; Vilar *et al*, 2007). Thus, new insight into the biology of these fascinating tumours could not only make prognostication easier, but also guide in the selection for the right treatment strategy, and contribute in the search for new drug targets. This last issue is of vital importance, as up till now, only surgery has the potential to cure patients with NET disease.

Prediction of the biological behaviour of NETs may be difficult based upon histological criteria alone (Wick, 2000; Stephenson, 2006). Well-differentiated NETs are easily recognised by routine tissue staining and conventional light microscopical (LM) examination, combined with immunohistochemical (IHC) detection of NE markers such as chromogranin A (CHGA) and synaptophysin (SYP). However, dealing with poorly differentiated tumours, it may be difficult to decide whether a tumour exhibits an NE character. Thus, new diagnostic markers are warranted.

In addition to classical NETs, it has been increasingly recognised that both mixed endocrine–exocrine malignant tumours, as well as NE differentiation in common epithelial cancers, may occur (Capella *et al*, 2000; Sørhaug *et al*, 2007). The picture is even more complex, as recent research has indicated that use of more sensitive methods such as the tyramide signal amplification technique, will identify more NE tumour cells than today's routine diagnostic procedures manage to do (Sørhaug *et al*, 2007). With respect to prognosis and treatment, the impact of such NE differentiation in epithelial cancers is mostly unknown.

To shed new lights into the biology of NETs, we have compared the gene expression pattern of a selection of NE tumour cells, with that of a group of non-NE tumour cells. By this approach, we have identified genes that are differentially expressed in NE *vs* non-NE tumour cells. We propose that some of the genes and their gene products may represent interesting new molecular factors with regard to tumorigenesis, prediction of prognosis and treatment response, as well as may represent novel therapeutic targets.

## Materials and methods

### Cell culture

Six NE and four non-NE cell lines were used in the gene expression analysis. All cell lines, except the BON cell line, were obtained from the American Type Culture Collection (ATCC, Manassas, VA, USA). BON cells (Evers *et al*, 1991) were a generous gift from Professor Kjell Öberg, Department of Medical Science, Uppsala University Hospital, Uppsala, Sweden, and cultured as described in Hofsli *et al* (2005). The six NE cell lines represent various NETs: neuroblastomas (SK-N-AS, SK-N-FI), bronchial carcinoids (NCI-H727, UMC-11), gastrointestinal carcinoid (BON), and medullary thyroid carcinoma (TT). The non-NE cell lines were colorectal adenocarcinomas (WiDr, SW480), lung adenocarcinoma (A-427) and glioblastoma (A-172). All these cell lines were cultured according to the requirements given by ATCC.

### Isolation of RNA

Cells were cultured in 75 cm^2^ culture flasks until 80% confluence, harvested and directly subjected to RNA isolation. Total RNA was isolated using RNeasy midi kit (Qiagen, Germantown, MD, USA), according to the manufacturer's instruction. Two independent biological experiments were performed with each cell line. The quality of the RNA was examined by use of Agilent 2100 Bioanalyzer (Agilent Technologies, Palo Alto, CA, USA). The samples were kept frozen at −80°C until further processing.

### Microarray hybridisation

Human cDNA arrays with 15 000 probes in duplicate were obtained from Norwegian Microarray Consortium, Oslo, Norway (http://www.microarray.no). These arrays were prepared using sequence-verified human genes (Research Genetics, Huntsville, AL, USA). Additional information of cDNA clone preparation and printing is described in detail within the platform GPL3313, of the Gene Expression Omnibus (http://www.ncbi.nlm.nih.gov/geo/query/acc.cgi?acc=GPL3313). Two negative controls and ten different cDNA spike-in controls from *Arabidopsis thaliana* (Stratagene SpotReporter, La Jolla, CA, USA) were included in all arrays. Total RNA (2 *μ*g) from the cell lines and from Universal Human Reference RNA (Stratagene, La Jolla, CA, USA), was reverse transcribed and labelled with Cy3- and Cy5-attached dendrimer, respectively, using the Genisphere 3DNA Array 350 Expression Array Detection kit (Genisphere, Montvale, NJ, USA), as described in the manufacturer's protocol and previously by us (Yadetie *et al*, 2003; Nørsett *et al*, 2004; Hofsli *et al*, 2005). To reduce the artefacts because of different sensitivity to photobleaching, the biologic replicates of each of the 10 cell lines were randomised by dye-swaps. The arrays were scanned separately by two wavelengths (532 and 633 nm) using ScanArray Express HT scanner (Packard BioScience, Billerica, MA, USA).

### Microarray data analysis

The microarray data were prepared according to the MIAME recommendations (Brazma *et al*, 2001). Image analysis was carried out using the GenePix Pro 4.1 software (Axon Instruments, Union City, CA, USA). All subsequent statistical analysis was performed using the statistical package R (R Development Core Team, 2004), and the LIMMA package from the Bioconduction project (Smyth, 2005). Flawed spots (manually examined) and spots with more than 40% saturated pixels in any channel were removed from the analyses. This resulted in the removal of 17–31% of spots for each array. To compensate for systematic errors each array was normalised using loess normalisation, and then scaled so that the log-transformed ratios had the same median absolute deviation (Yang *et al*, 2002a, 2002b). Further analyses were based on these normalised log-transformed ratios for each duplicate gene for the 20 microarrays.

To assess the difference between the NE *vs* non-NE tumour cells for each gene, tests for differential expression were performed using moderated *t*-tests based on duplicated spots, as implemented in the Limma R package of Smyth *et al* (2005). This is based on empirical Bayes analysis, where the power of the tests is improved by replacing gene-specific variance estimates with estimates found by borrowing strength from data on the remaining genes. The proportion of truly differentially expressed genes was estimated using the convex decreasing density estimator of Langaas (2005), and the false discovery rate (FDR) was estimated using the method of Storey (2002), with the estimated proportion of truly differentially expressed genes found above inserted.

Cluster analysis was performed as an aid to display the results in a graphical manner. The analysis was performed on the normalised log ratios taking the median over duplicate spots for each gene, and the mean over the dye-swapped replicates. Hierarchical cluster analysis was based on Pearson correlation and the distance between the clusters was both computed using the average- and complete linkage. In addition clustering using the K-means algorithm (using two clusters) was also performed on a selection of the most differentially expressed genes.

### Real-time qRT–PCR

cDNA synthesis was performed with 500 ng total RNA in a 10-*μ*l reaction containing 1 × PCR buffer II, 5 mM MgCl_2_, 500 *μ*M each dNTP, 2.5 *μ*M. Oligo d(T)16 primer, 0.4 U/*μ*l RNase inhibitor and 2.5 U/*μ*l MuLV Reverse transcriptase (Applied Biosystems, Mannheim, Germany). cDNA synthesis was performed at 10 min at 25°C followed by 1 h at 48°C and 5 min at 95°C. Design of PCR primers and probes was performed using Primer3 (version 0.2, online software) (http://frodo.wi.mit.edu/cgi-bin/primer3/primer3_www.cgi) and the mRNA sequences obtained using the NCBI RefSeq accession numbers of the respective genes (Table 1). All primers and probes were delivered from Eurogentec, Seraing, Belgium, and had an optimal annealing temperature of 56 and 68°C, respectively. TaqMan real-time PCR was performed with 1 × Quantitect Probe PCR Master Mix (Qiagen, Germantown, MD, USA), 400 nM of each primer, 200 nM TaqMan Probe (Eurogentec) or sybergreen and cDNA equivalent to 62.5 ng total RNA in a total reaction volume of 25 *μ*l. The Real-Time PCR was performed in Stratagene's Mx3000P Real Time PCR system; 15 min at 95°C, 40 thermal cycles of 15 s at 94°C, 30 min at 56°C and 30 s at 76°C. Each sample was measured in triplicate. A negative control without the cDNA template was included, and contamination by genomic DNA was ruled out by performing PCR analysis on template where reverse transcriptase had been omitted in the RT reactions. GAPDH was run in parallel as controls to monitor RNA integrity and to be used for normalisation. Fold induction of gene expression level was estimated by the ΔΔ*C*_t_-method, where: Fold change=2^−ΔΔC*t*^ and ΔΔ*C*_t_=(*C*_tGOI_−*C*_tGAPDH_)_untreated_−(*C*_tGOI_−*C*_tGAPDH_)_treated_ (Livak and Schmittgen, 2001). This was accomplished by using the same universal human reference RNA in both the microarray and the real-time RT–PCR analysis; 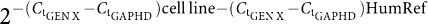


### Western blot

Whole cell lysates were prepared from 5–7 × 10^6^ cells which were washed two times in PBS, scraped and harvested directly in 2000 *μ*l SDS-sample buffer (62.5 mM Tris-HCl, pH 6.8; 8.7% glycerol; 2% w/v SDS; 5% v/v 2-*β* mercaptoethanol; 0.09% w/v bromophenol blue). Viscosity was reduced by drawing the suspension through a 21-G needle, cell debris were removed by centrifugation (15 000 g, 10 min), and the supernatant was stored at −80°C. Each extract (15 *μ*l) was boiled and separated on an SDS 10% polyacrylamide gel (running buffer: 25 mM Tris-HCl, pH 8.3; 190 mM glycine, 0.1% w/v SDS) before electrotransfer onto Hybond-P membranes (Amersham Pharmacia Biotech, Pittsburgh, PA, USA). The transfer was performed in 25 mM Tris-HCl, 190 mM glycine and 20% methanol, pH 8.3, for 1 h at 175 mA. The membranes were treated with 5% nonfat dry milk (Nestlé, Vevey, Switzerland) in TBS (50 mM Tris-HCl, pH 7.5 and 150 mM NaCl) for 1 h at room temperature and incubated with primary antibodies diluted (1 : 500–1 : 1000) in TBS with 1% BSA and 0.05% Tween 20 for 2 h, 20°C. The blots were then incubated with peroxidase-conjugated secondary antibodies (1 : 1000) in TBS with 1% BSA and 0.05% Tween 20 for 1 h at room temperature. After washing (4 × 15 min in TBS with 0.05% Tween 20), binding of secondary antibodies was visualised by the ECL-detection system (Amersham) before they were digitally exposed with the KODAK Image Station 2000R (Kodak, Rochester, NY, USA) for 5 min. GAPDH levels were used to verify protein loading.

The following antibodies were used: mouse anti-human GAPDH (1 : 1000) (Abcam, Cambridge, UK), mouse anti-human PRDX2 (1 : 1000) (Abcam), rabbit anti-human HPN (1 : 1000) (Cayman, Michigan, USA), rabbit anti-human SCG2 (1 : 500) (Abcam) and a secondary antibody conjugated to horseradish peroxidase (Pierce, Rockford, IL, USA).

### Immunohistochemical and ultrastructural examinations

For IHC investigations, cell pellet was conventionally fixed in 10% neutral formalin, dehydrated, and embedded in paraffin. Sections, about 4–5 micron thick, were employed for the IHC examinations, using the Vectastain ABC kit (Vector Lab., Burlingame, CA, USA), and/or Tyramide signal amplification technique (NEN LifeScience Products, Boston, MA, USA), as previously described (Qvigstad *et al*, 1999). Chromogranin A antiserum (1 : 500) was provided by Incstar (Stillwater, MN, USA), monoclonal mouse antisynaptophysin antiserum (1 : 20) by Dako (Glostrup, Denmark), anti rat neuron-specific enolase (1 : 500) by Polysciences (Warrington, PA, USA), and antineurofilament M (1 : 4000) by Fitzgerald (MA, USA).

For the electron microscopic (EM) investigations, the pellet was fixed in 2% neutral glutaraldehyde, post-fixed in 2% osmium tetroxide, contrasted with 1% lead citrate and 4% uranyl acetate, and conventionally embedded in Epon. Finally, conventional ultra thin sections were cut and analysed by means of our transmission EMs (JEOL 100CX and Phillips SEI Tecnai 12).

## Results

### Confirmation of the NE character

To confirm the NE and non-NE character of the cell lines, respectively, IHC and EM investigations were performed in addition to conventional LM examination. The employed NE cell lines (NCI-H727, UMC-11, SK-N-AS, SK-N-FI, TT, BON) encompass NE features with the expression of CHGA and SYP as the confined NE marker. The four cell lines known to be of non-NE character (WiDr, A-172, A-427, SW480), showed no staining with CHGA and SYP (data not shown). In addition, the cells were examined for the expression of ENO2 (enolase 2/neuron-specific enolase), an NE marker thought to be less specific than the conventional NE markers CHGA and SYP. All the presumed NE cell lines showed positive immunoreactivity to enolase 2, and this was also the case for the non-NE cell lines A-427 and SW480 (data not shown). EM investigations demonstrated occurrence of typical NE secretion granules in all the NE tumour cells, but not in any of the non-NE tumour cells, thus confirming the predefined NE/non-NE characteristics of the cell lines used.

### Genes differentially expressed in NE *vs* non-NE tumour cells

Having confirmed the NE and non-NE character of the cell lines, respectively, we performed transcript profiling by cDNA microarray analysis in an effort to identify new NE-specific genes, and by this, get more insight into the biology of NETs.

By using the convex decreasing density estimator for the proportion of true null hypotheses as presented in Langaas (2005), we expect 5.5% of the genes studied to be differentially expressed in NE *vs* non-NE cells. The 200 most significant genes (*P*-value 0.008/FDR 0.49) in the comparison of the NE *vs* non-NE tumour cell groups are sequence verified, and 153 genes are given as Supplementary Information in the gene expression omnibus (GEO) GSE4328.

**Figure 1:**
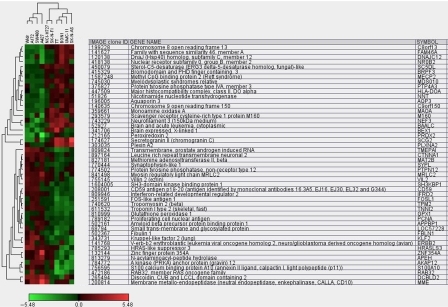
Hierarchical clustering analysis. A hierarchical clustering algorithm was used to cluster experimental samples on the basis of similarities of gene expression. Relationships between the experimental samples are summarised as dendrograms, in which the pattern and length of the branches reflect the relatedness of the samples (NE *vs* non-NE). Data are presented in a matrix format: each row represents a cDNA clone (identified with UniGene gene symbol, name and IMAGE clone id) and each column an individual mRNA (average gene expression log ratio) sample of NE (BON, TT, SK-N-AS, SK-NFI, NCI-H727, UMC-11) and non-NE (WiDr, A-172, SW480, A-427) cells. The results presented represent the ratio of hybridisation of fluorescent cDNA probes prepared from each experimental mRNA sample to a reference mRNA sample. These ratios (log) are a measure of relative gene expression in each experimental sample and were depicted according to the colour scale shown at the bottom.

Based on information from the GO annotation database and literature search, these genes are displayed with the log ratio and biological processes in which they are likely to be involved. The up- and downregulated genes range from log_2_ 5.87 to −2.92, respectively. The 70 most highly up- and downregulated genes, are shown in Table 2. A hierarchical cluster analysis of the 48 most significantly differentially expressed genes (*P*-value 0.0014/FDR 0.2823) are shown in Figure 1.

The three most highly overexpressed genes: *SCG3* (26.6 fold), *SCG2* (15.3 fold) and *DDC* (9.6 fold) (Table 2), have previously been shown to be linked to NE tumour biology, thus confirming the reliability of our study design. SCG3 and SCG2 are both members of the chromogranin–secretogranin family of NE secretory, acidic glycoproteins (Taupenot *et al*, 2003), and DDC has more recently been shown to be expressed in various NETs (Uccella *et al*, 2006). Furthermore, the high expression of *MAOA* in our study, support previous findings of high expression of monoaminoxidase A in various NETs (Örlefors *et al*, 2003).

NETs in general are relatively slow growing tumours with a less invasive character than many epithelial cancers. Several genes thought to play a role in the processes of invasion, tumour progression and metastasis (*MME*, *STAT3*, *DCBLD2*, *S100A10*, *CD9*, *S100A8*) were highly downregulated in the NE *vs* the non-NE tumour group (Table 2 and Supplementary Information). The three most highly downregulated genes in our study were *MME* (0.12 fold), *STAT3* (0.13) and *DCBLD2* (0.14 fold). Our results also point to differences in expression of several genes thought to be involved in the process of tumorigenesis (*BEX1*, *TMEPAI*, *FOSL1*, *RAB32*, *ERBB2*) (Table 2 and Supplementary Information). Well-differentiated NETs are in general relatively insensitive to various chemotheurapeutic drugs, and thus it is interesting to note variations between the two groups in the expression of genes known to be involved in the process of drug resistance (*STAT3*, *PRXD2 ABCC6*, *GSTP1*) (Table 2 and Supplementary Information). Nearly 50% of the upregulated, and 16% of the downregulated genes are in the GO database defined as having an unknown function (Supplementary Information).

### Validation by real-time qRT–PCR

**Figure 2:**
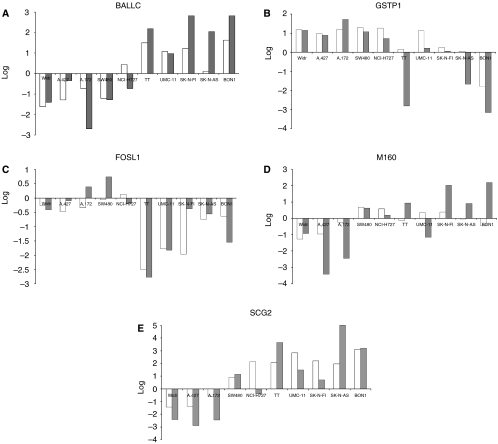
RT–PCR confirmation of microarray results. A selection of genes (**A**–**E**) was also analysed by semi quantitative real-time RT–PCR (grey), and compared to the respective ratios of the microarray analysis (white). The two methods correlated at 9/10 cell lines at best, and the lowest correlated at 6/10 cell lines. *Y* axis shows the log-transformed ratio of both the microarray and the RT–PCR, based on the fold change ratios and the delta–delta *C*_t_ calculation, respectively.

To validate the microarray results, we performed real-time quantitative RT–PCR analysis of five selected genes using the same RNA samples as those used in the microarray analysis. The selection of the genes (*BAALC*, *SCG2*, *GSTP1*, *FOSL1*, *M160*) were based upon a combination of *P*-value, differential expression, and biological function. In general, 70% of the genes found to be differentially expressed in the microarray study were confirmed by RT–PCR (Figure 2). This seems to be in accordance with previous studies using cDNA arrays (Kothapalli *et al*, 2002; Hofsli *et al*, 2005), and underlines the need to verify microarray data by additional methods.

### Protein expression analysis

**Figure 3:**
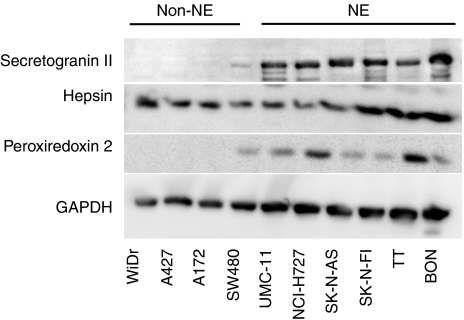
Western blot. Western blot analysis was performed on cell lines (NE and non-NE) with the antibodies against secretogranin II, hepsin, peroxiredoxin 2 and GAPDH. Cells were harvested and prepared as described in Materials and methods.

To investigate whether the difference in gene expression level was followed by a similar expression pattern at the protein level, we first performed western blot analysis of cell lysates. The selection of gene products analysed (secretogranin II, peroxiredoxin 2, hepsin) was based upon a combination of the expression level found in the microarray analysis, biological relevance, and availability of antibodies. As seen in Figure 3, the protein expression of the NE marker secretogranin II, correlated well with the gene expression level of *SCG2* found in the microarray analysis (15-fold upregulated)(Table 2), and in the real-time RT–PCR analysis (Figure 2). All the NE tumour cell lines express a high level of SCG2, whereas the expression level in the non-NE cell group is almost undetectable. Hepsin (2.8 fold upregulated in the microarray analysis) was found to be expressed in all cell lines and without any significant difference in NE *vs* non-NE cells (Figure 3). Thus, hepsin is ruled out as a possible new diagnostic marker of NET disease. On the contrary, the level of peroxiredoxin 2 expression (5 fold upregulated in the microarray analysis) was significantly different in the two groups (Figure 3). Peroxiredoxin 2 was clearly detectable in the NE cell line group, but almost undetectable in the non-NE cell group, thus pointing out peroxiredoxin 2 as an interesting new NE biomarker. The difference in secretogranin II and peroxiredoxin 2 expression was also confirmed by IHC analysis (data not shown).

In addition to secretogranin II and peroxiredoxin 2, our study points to NEFM as another interesting candidate marker of NET disease. *NEFM*, which was upregulated by a factor of 7.7 in the microarray analysis (Table 2), was by IHC shown to be expressed only in the NE tumour cells group (data not shown).

## Discussion

Although last year's genomic and proteomic research have uncovered some genes and gene products thought to have an important function in the context of NE tumour biology (Hofsli, 2006), still much is unknown concerning which factors that are important with regard to the causes and behaviours of NET diseases. The results of this study contribute to an increased insight into the biology of these tumours, by identifying genes that are differentially expressed in NE tumour cells as compared with non-NE tumour cells. We believe that some of these genes and gene products represent interesting candidates in the search for new prognostic, predictive and therapeutic markers. The study also point to genes that may play a role in the tumorigenesis of NETs.

The three most highly overexpressed genes in the NE *vs* the non-NE tumour cell group (*SCG3*, *SCG2* and *DDC*) (Table 2), have all previously been described in the context of NE tumour biology, thus confirming the reliability of our study design. Although secretogranin II and one of its split product (Taupenot *et al*, 2003; Guillemot *et al*, 2006) have been shown to be expressed in various types of NETs, investigations of the expression of secretogranin III in NETs have so far not been reported. The enzyme dopa decarboxylase (DDC)(catecholamine biosynthesis) has more recently been shown to be expressed in various NETs, such as bronchial carcinoids and poorly differentiated NE carcinomas of the lung (Uccella *et al*, 2006). It has also been shown to be a marker of neuroblastoma in children (Bozzi *et al*, 2004), and of NE differentiation in prostate carcinoma (Wafa *et al*, 2007). Another gene known to be involved in catecholamine metabolism, *MAOA* (Toninello *et al*, 2006), was also identified as highly expressed in the NET group (Table 2), a finding that was confirmed by IHC analysis (not shown). This supports previous findings demonstrating a high expression of MAOA in gastroenteropancreatic (GEP) tumours (Örlefors *et al*, 2003). To conclude, we believe that SCG3, SCG2 and DDC could represent useful additional biomarkers in NET diseases, and that they perhaps should be implemented in the standard diagnostic panel of NE biomarkers. Furthermore, measurement of MAOA activity may, as recently shown in a baboon model, aid in understanding the pathophysiology of NETs (Murthy *et al*, 2007).

In addition to these above-mentioned potentially important NET biomarkers, our study points to NEFM, PRDX2, and CLDN4 as other interesting candidate markers of NET disease. The finding of an upregulation of the NEFM gene (a marker of neuronal differentiation) is in accordance to findings by Perez *et al* (1990), who found NEFM expression in a subset of pancreatic islet cell and rectal carcinoid tumours, although rarely in ileojejunal carcinoid tumours. Thus, the message brought from our study and that of Perez is, that neurofilament subtyping could well become a potential diagnostic tool with regard to NETs.

Also the antioxidant enzyme peroxiredoxin 2 (PRXD2) (antiapoptosis) was highly upregulated in the NE tumour cell group. PRXD2 was previously shown to be elevated in several human cancers, to confer resistance to chemo- and radiation therapy, and to promote tumour progression and metastasis (Lee *et al*, 2007). The tight junction protein claudin 4 (CLDN4) is also frequently overexpressed in several cancers, and is thought to represent a promising target for cancer detection, diagnosis, and therapy (Morin, 2005; Kominsky *et al*, 2007). A loss of claudin 4 expression at the invasive front in colorectal cancer correlates with cancer invasion and metastasis (Ueda *et al*, 2007), and thus the finding in our study of a rather high level of *CLDN4* in the NE tumour group, may reflect NETs in general lower malignant phenotype. However, our results are in contrast to that of Moldvay *et al* (2007), who more recently have demonstrated that a majority of bronchial carcinoids express a lower level of CLDN4 than other histological types of primary bronchial cancers.

Several of the differentially expressed genes turned out to have unknown functions (Supplementary Information; GEO GSE4328). We focused on *BAALC* (brain and acute leukaemia, cytoplasmic), as a high mRNA transcript level of this gene has been found in tissues of neuroectodermal origin (Tanner *et al*, 2001), and has been shown to be an independent adverse prognostic factor in various acute leukaemias (Marcucci *et al*, 2005; Baldus *et al*, 2007). The high expression of *BAALC* found in the microarray analysis (Table 2), was confirmed by real-time PCR analysis (Figure 2).

Our results also point to differences in expression of several genes thought to be involved in the process of tumorigenesis (*BEX1*, *TMEPAI*, *FOSL1*, *RAB32*, *ERBB2*) (Table 2; Supplementary Information). One interesting find is the high expression of the novel BEX1 gene (brain expressed, X-linked 1) in the NE tumour cell group (Table 2). Previous studies have revealed a high expression of this gene in brain, but also in peripheral organs such as liver, pancreas, testis, and ovary (Yang *et al*, 2002a, 2002b; Alvarez *et al*, 2005). It has more recently been suggested that *BEX1* may play a role as a tumour suppressor in malignant glioma (Foltz *et al*, 2006). A very low expression was observed for the TMEPAI gene (Table 2), which is involved in androgen receptor signaling, and is proposed to play a role in prostate tumorigenesis (Xu *et al*, 2003). TMEPAI has been shown to be overexpressed in various solid tumours, probably because of abnormal activation of the EGF pathway (Giannini *et al*, 2003). Also the oncogenic transcription factor *FOSL1*, was downregulated in the NE tumour cell group. FOSL1 is upregulated in several solid cancers, and is becoming a new target for cancer intervention (Young and Colburn 2006). The ras family member *RAB32*, has been proposed to represent a component of the oncogenic pathway of microsatellite instability-high gastrointestinal adenocarcinomas (Shibata *et al*, 2006). In our study, *RAB32* was highly downregulated in the NE *vs* the non-NE group. Also the ERBB2 gene expression level was significantly lower in the NE tumour cell group than in the non-NET group. The expression level of this member of the oncogenic EGF receptor family, has previously been reported as a variable in various NETs (*cf.* Hofsli, 2006). So far, there is no strong evidence that *ERBB2* amplification/overexpression could play an important role in NET pathogenesis, or that it could be a potential target for treatment, as is the case in various epithelial cancers (Hsieh and Moasser 2007). To conclude, our study is the first to reveal the expression pattern of *BEX1*, *TMEPAI*, *FOSL1,* and *RAB32* in NE tumour cells, and we believe that they represent interesting novel candidates in the context of NET tumorigenesis.

A hallmark of NETs in general, are that they are relatively slow growing and less invasive in character. Thus, its interesting to note that several genes thought to play a role in the processes of invasion, tumour progression and metastasis (*MME*, *STAT3*, *DCBLD2*, *S100A10*, *CD9*, *S100A8*) were highly downregulated in the NE *vs* the non-NE tumour group (Table 2). The most highly downregulated gene was MME. A loss or decrease in MME has been reported in a variety of malignancies, and reduced expression results in the accumulation of higher peptide concentrations that could mediate neoplastic progression (Sumitomo *et al*, 2005). Loss of this endopeptidase also leads to AKT1 (protein kinase B) activation, and contributes to the clinical progression of prostate cancer (Osman *et al*, 2006). STAT3 (the signal-transducer and activator –of transcription 3) is thought to play an important role in both tumorigenesis and tumour progression, and is often constitutively activated in tumour cells (Aggarwal *et al*, 2006). Thus, inhibitors of STAT3 activation have potential for both prevention and therapy of cancer (Huang, 2007). In lung cancer, *DCBLD2* has been shown to be highly upregulated in the cell line NCI-H460-LNM35, in association with its acquisition of metastatic phenotype, and also upregulated in high frequency in metastatic lesions from lung cancers (Koshikawa *et al*, 2002). It is also shown that *DCBLD2* may play a role in cell motility (Nagai *et al*, 2007), and thus it is suggested that this novel gene may become a target of therapy to inhibit metastasis of lung cancers.

The plasminogen receptor S100A10 is found overexpressed in many cancer cells, and seems to play an important role in cancer cell invasiveness and metastasis (Kwon *et al*, 2005). RNA interference-mediated downregulation of S100A10 gene expression in colorectal cancer cells, has been shown to result in a complete loss in plasminogen-dependent cellular invasiveness (Zhang *et al*, 2004). More recently it has been shown by IHC analysis that S100A10 expression in thyroid neoplasms contributes to the aggressive characteristic of anaplastic carcinoma (Ito *et al*, 2007). To conclude, the very low levels of various genes known to be involved in the processes of invasion, tumour progression and metastasis could perhaps reflect the in general more slow growing and less invasive character of NETs.

In addition to the already mentioned *STAT3* and *PRXD2*, other genes that have been linked to the phenomenon of drug resistance, were identified as differentially expressed (*ABCC6*, *GSTP1*). Well-differentiated NETs are in general relatively insensitive to various chemotheurapeutic drugs. Thus, it is interesting to note that our study reveals a relatively high expression of *ABCC6* (ATP-binding cassette, subfamily C (CFTR/MRP), member 6), one member of the MRP subfamily involved in multi-drug resistance (Beck *et al*, 2005). Endocrine G-cells in the stomach has been shown to express high level of ABCC6 (Beck *et al*, 2005). However, our study is the first to report *ABCC6* expression in NE tumour cells. The antiapoptosis gene GSTP1, was highly downregulated in the NET group (Table 2). In prostate cancer, the loss of expression of *GSTP1* is the most common genetic alteration reported (Meiers *et al*, 2007). A comprehensive survey of GSTP1 expression in NETs has so far not been performed, but one study has been undertaken, showing that the expression of this drug-resistant protein is significantly lower in large cell NE carcinoma of the lung than in the other more common histological types of lung cancer (Okada *et al*, 2003).

In conclusion, the results of our study add new important lights into the understanding of NE tumour biology, by identifying genes differentially expressed in NE as compared with non-NE tumour cells. In addition to potential new diagnostic markers (*SCG2*, *SCG3*, *DDC*, *MAOA*, *NEFM*, *CLDN4*, *PEROX2*), genes critical in the processes of tumour invasion, progression and metastasis (*MME*, *STAT3*, *DCBLD2*, *S100A10*, *CD9*, *S100A8*), tumorigenesis (*BEX1*, *TMEPAI*, *FOSL1*, *RAB32*) and drug-resistance (*ABCC6*, *GSTP1*) were identified, as well as several genes with hitherto unknown functions.

## Figures and Tables

**Table 1:** Primers and probes

**Symbol/gene Bank Accession No.**	**Sequence (5′ → 3′)**	**Forward reverse probe**	**Product length**
BAALC	actgcccatggcatgtct	S	66
NM_024812	tccaggcagatgaggagc	AS	
	tgggaggtgtctgtgaagcagtca	Probe	
FOSL1	accctcagtacagccccc	F	81
NM_005438	aaggccttcgacgtaccc	AS	
	aaccccggccaggagtcatc	Probe	
GSTP1	tgcctatacgggcagctc	F	102
NM_000852	cccatagagcccaagggt	AS	
	aagttccaggacggagacctcacc	Probe	
SCG2	tggctgaagcaaagaccc	F	75
NM_003469	cagccccagagatgagga	AS	
	tggagcagccctgtctcttatccc	Probe	
M160	tctatcacgacggcttct	F	174
NM_174941	ccattcctgtgcagttca	AS	
	aatgccacggtctctgctcacttt	Probe	
GAPD		F	
NM_002046		AS	
		Probe	

Genes, primers, and probe sequences of selected genes for confirmation studies. The length, product length, and orientation are given here.

**Table 2:** Differentially expressed genes in NE *vs* non-NE tumour cells

**Gene symbol**	**Gene name**	**UGCluster**	**Ratio**
*Upregulated*			
SCG3	Secretogranin III	Hs.232618	26.56
SCG2	Secretogranin II (chromogranin C)	Hs.516726	15.29
DDC	Dopa decarboxylase (aromatic L-amino acid decarboxylase)	Hs.359698	9.65
BAALC	Brain and acute leukaemia, cytoplasmic	Hs.533446	7.78
NEF3	Neurofilament 3	Hs.458657	7.66
C8orf13	Chromosome 8 open reading frame 13	Hs.124299	7.27
BEX1	Brain expressed, X-linked 1	Hs.334370	6.76
RAPGEF5	Rap guanine nucleotide exchange factor (GEF) 5	Hs.174768	6.01
PLXNA2	Plexin A2	Hs.497626	5.88
	Hypothetical LOC90024	Hs.534513	5.30
MGC17299	Hypothetical protein MGC17299	Hs.104476	5.11
PRDX2	Peroxiredoxin 2	Hs.432121	5.04
M160	Scavenger receptor cysteine-rich type 1 protein M160	Hs.49636	5.01
DNAJC12	DnaJ (Hsp40) homologue, subfamily C, member 12	Hs.260720	4.50
MAOA	Monoamine oxidase A	Hs.183109	4.48
FAM46A	Family with sequence similarity 46, member A	Hs.10784	4.29
FNDC5	Fibronectin type III domain containing 5	Hs.524234	4.11
CLDN4	Claudin 4	Hs.520942	3.96
CACNA1H	Calcium channel, voltage-dependent, *α* 1H subunit	Hs.459642	3.95
ITGA10	Integrin, *α* 10	Hs.158237	3.88
HLA-DOA	Major histocompatibility complex, class II, DO *α*	Hs.351874	3.70
CNTN1	Contactin 1	Hs.143434	3.65
NR0B2	Nuclear receptor subfamily 0, group B, member 2	Hs.427055	3.57
TAGLN3	Transgelin 3	Hs.169330	3.45
PEG10	Paternally expressed 10	Hs.147492	3.37
EGLN3	Egl nine homologue 3 *(C. elegans)*	Hs.135507	3.36
MBP	Myelin basic protein	Hs.551713	3.36
ABCC6	ATP-binding cassette, subfamily C (CFTR/MRP), member 6	Hs.13188	3.26
SFMBT2	Scm-like with four mbt domains 2	Hs.407983	3.21
C9orf150	Chromosome 9 open reading frame 150	Hs.445356	3.17
	CDNA FLJ37828 fis, clone BRSSN2006575	Hs.123119	3.15
HIPK2	Homeodomain interacting protein kinase 2	Hs.397465	3.10
	CDNA FLJ45289 fis, clone BRHIP3002363	Hs.556782	3.08
C17orf28	Chromosome 17 open reading frame 28	Hs.11067	3.05
C3orf14	Chromosome 3 open reading frame 14	Hs.47166	2.94
LIMD1	LIM domains containing 1	Hs.193370	2.92
HPN	Hepsin (transmembrane protease, serine 1)	Hs.182385	2.82
MDS010	x 010 protein	Hs.231750	2.77
MS4A1	Membrane-spanning 4-domains, subfamily A, member1	Hs.438040	2.75
NAPB	*N*-ethylmaleimide-sensitive factor attachment protein, *β*	Hs.269471	2.69
PBX1	Pre-B-cell leukaemia transcription factor 1	Hs.493096	2.63
APG4A	APG4 autophagy 4 homologue A	Hs.8763	2.60
ARHGAP26	Rho GTPase-activating protein 26	Hs.293593	2.56
GAB2	GRB2-associated binding protein 2	Hs.429434	2.53
AQP3	Aquaporin 3	Hs.234642	2.45
MGC4645	Hypothetical protein MGC4645	Hs.395306	2.44
PTP4A3	Protein tyrosine phosphatase type IVA, member 3	Hs.43666	2.40
TP53I11	Tumour protein p53-inducible protein 11	Hs.554791	2.36
	Clone IMAGE:121214 mRNA sequence	Hs.283883	2.36
C14orf132	Chromosome 14 open reading frame 132	Hs.6434	2.33
SC5DL	Sterol-C5-desaturase	Hs.287749	2.29
CENTB5	Centaurin, *β* 5	Hs.535257	2.15
ARHGAP5	Rho GTPase-activating protein5	Hs.525287	2.13
KIAA0924	KIAA0924 protein	Hs.560561	2.10
C6orf1	Chromosome 6 open reading frame 1	Hs.381300	2.10
SGTB	Small glutamine-rich tetratricopeptide repeat (TPR)	Hs.287971	2.05
NNT	Nicotinamide nucleotide transhydrogenase	Hs.482043	2.05
BRPF3	Bromodomain and PHD finger containing, 3	Hs.520096	1.99
TBC1D16	TBC1 domain family, member 16	Hs.369819	1.98
IRF2BP2	Interferon regulatory factor 2-binding protein 2	Hs.350268	1.94
DKFZp434H2226	LMBR1 domain containing 2 (DKFZp434H2226)	Hs.294103	1.93
CALM1	Calmodulin 1 (phosphorylase kinase, delta)	Hs.282410	1.82
C6orf209	Chromosome 6 open reading frame 209	Hs.271643	1.79
ZCCHC3	Zinc finger, CCHC domain containing 3	Hs.28608	1.78
IRS2	Insulin receptor substrate 2	Hs.442344	1.76
RGS18	Regulator of G-protein signalling 18	Hs.440890	1.70
SCFD1	Sec1 family domain containing1	Hs.369168	1.69
TCEAL8	Transcription elongation factor A (SII)-like 8	Hs.389734	1.67
SEC23B	Sec23 homologue B (S. cerevisiae)	Hs.369373	1.59
MECP2	Methyl CpG-binding protein 2	Hs.200716	1.59
			
*Downregulated*			
MME	Membrane metallo-endopeptidase	Hs.307734	0.12
STAT3	Signal transducer and activator of transcription 3 (acute-phase response factor)	Hs.463059	0.13
MEOX1	Homeobox protein MOX-1	Hs.438	0.14
TMEPAI	Transmembrane, prostate androgen induced RNA	Hs.517155	0.15
RAB32	RAB32, member RAS oncogene family	Hs.287714	0.17
KLF2	Kruppel-like factor 2 (lung)	Hs.107740	0.17
ZNF354A	Zinc finger protein 354A	Hs.484324	0.17
LOC255104	Hypothetical protein LOC255104	Hs.466729	0.18
S100A10	S100 calcium-binding protein A10 (annexin II ligand, calpactin I, light polypeptide (p11))	Hs.143873	0.18
DCBLD2	Discoidin, CUB and LCCL domain containing 2	Hs.203691	0.18
HSPC016	Hypothetical protein HSPC016	Hs.356440	0.20
USP4	Ubiquitin-specific peptidase 4 (proto-oncogene)	Hs.77500	0.21
GSTP1	Glutathione *S*-transferase pi	Hs.523836	0.22
AKAP12	A kinase (PRKA) anchor protein (gravin) 12	Hs.371240	0.23
MGC7036	Hypothetical protein MGC7036	Hs.488173	0.24
ZF	HCF-binding transcription factor Zhangfei	Hs.535319	0.25
AMOTL2	Angiomotin like 2	Hs.426312	0.25
LMO2	LIM domain only 2	Hs.34560	0.25
CETN2	Centrin, EF-hand protein, 2	Hs.82794	0.27
RUNX1	Runt-related transcription factor 1 (acute myeloid leukaemia 1; aml1 oncogene)	Hs.149261	0.27
HRASLS3	HRAS-like suppressor 3	Hs.502775	0.28
APEH	*N*-acylaminoacyl-peptide hydrolase	Hs.517969	0.28
ERBB2	V-erb-b2 erythroblastic leukaemia viral oncogene homologue 2, neuro/glioblastoma derived oncogene homologue	Hs.446352	0.28
YAP1	Yes-associated protein 1, 65kDa	Hs.503692	0.31
CD9	CD9 antigen (p24)	Hs.114286	0.31
TNNI2	Troponin I, skeletal, fast	Hs.523403	0.32
FBLN1	Fibulin 1	Hs.24601	0.32
S100A8	S100 calcium binding protein A8 (calgranulin A)	Hs.416073	0.33
LOC91614	Novel 58.3 KDA protein	Hs.280990	0.34
CTSL	Cathepsin L	Hs.418123	0.34
PRPS2	Phosphoribosyl pyrophosphate synthetase 2	Hs.104123	0.35
TMSB10	Thymosin, *β* 10	Hs.446574	0.37
TPM2	tropomyosin 2 (*β*)	Hs.300772	0.37
SH3KBP1	SH3-domain kinase-binding protein 1	Hs.444770	0.38
FOSL1	FOS-like antigen 1	Hs.283565	0.39
ODC1	Ornithine decarboxylase 1	Hs.467701	0.39
MRLC2	Myosin regulatory light chain MRLC2	Hs.464472	0.39
LOC57228	Hypothetical protein from clone 643	Hs.558523	0.39
CD164	CD164 antigen, sialomucin	Hs.520313	0.41
CAMK1	Calcium/calmodulin-dependent protein kinase I	Hs.434875	0.41
RPA3	Replication protein A3, 14kDa	Hs.487540	0.41
VIL2	Villin 2 (ezrin)	Hs.487027	0.42
IFRD2	Interferon-related developmental regulator 2	Hs.315177	0.42
NLGN2	Neuroligin 2	Hs.26229	0.43
CD59	CD59 antigen p18-20	Hs.278573	0.43
ZBTB4	Zinc finger and BTB domain containing 4	Hs.35096	0.45
TXNRD1	Thioredoxin reductase 1	Hs.337766	0.46
MAT2B	Methionine adenosyltransferase II, *β*	Hs.54642	0.46
BMP1	Bone morphogenetic protein 1	Hs.1274	0.46
HRB2	HIV-1 rev binding protein 2	Hs.205558	0.47
APPBP1	Amyloid *β* precursor protein binding protein 1, 59kDa	Hs.460978	0.48
CTNNA1	Catenin (cadherin-associated protein), *α* 1, 102kDa	Hs.445981	0.49
COMMD6	COMM domain containing 6	Hs.508266	0.51
MAP4	Microtubule-associated protein4	Hs.517949	0.51
PSMD12	Proteasome (prosome, macropain) 26S subunit, non-ATPase, 12	Hs.4295	0.51
PLP2	Proteolipid protein 2 (colonic epithelium-enriched)	Hs.77422	0.52
GPX1	Glutathione peroxidase 1	Hs.76686	0.52
SYPL	Synaptophysin-like protein	Hs.80919	0.54
PICALM	Phosphatidylinositol-binding clathrin assembly protein	Hs.163893	0.56
PTPN12	Protein tyrosine phosphatase, non-receptor type 12	Hs.61812	0.56
PSMA5	Proteasome (prosome, macropain) subunit,*α* type5	Hs.485246	0.58
ST13	Suppression of tumorigenicity 13 (colon carcinoma) (Hsp70 interacting protein)	Hs.546303	0.58
SR140	U2-associated SR140 protein	Hs.529577	0.58
PAWR	PRKC, apoptosis, WT1, regulator	Hs.406074	0.59
EGR3	Early growth response 3	Hs.534313	0.60
HNRPH1	Heterogeneous nuclear ribonucleoprotein H1 (H)	Hs.202166	0.60
GLTSCR2	Glioma tumour suppressor candidate region gene 2	Hs.421907	0.61
PCNA	Proliferating cell nuclear antigen	Hs.147433	0.61
PSMB3	Proteasome (prosome, macropain) subunit, *β* type, 3	Hs.82793	0.61
EMR3	Egf-like module containing, mucin-like, hormone receptor-like 3	Hs.295626	0.62

Genes significantly (*P*<0.007) up- or downregulated in the neuroendocrine cell lines compared with the non-neuroendocrine cell lines. The first half of the table shows downregulated genes whereas the last part of the table shows the upregulated genes. The genes are all given with unigene cluster id's, official gene name and symbols in addition to their respective ratio (NE *vs* non-NE).
